# Supplementary material for: The export receptor Crm1 forms a dimer to promote nuclear export of HIV RNA
Source: eLife. 2014 Dec 8;3:e04121. doi: 10.7554/eLife.04121 (PMC4360530; doi:10.7554/eLife.04121)
Supplement: Supplementary file 1. — Plasmids for protein and RNA expression. DOI: http://dx.doi.org/10.7554/eLife.04121.013 [file elife04121s001.docx]

| Plasmids for protein and RNA expression | | | |
| --- | --- | --- | --- |
| Name | Description | Reference | |
| pHG-Rev | HXB3 Rev | | 36 |
| pHG-Rev M10 | Site directed mutagenesis of pHG-Rev. | This study | |
| pET19b-hCrm1 | Amplified from *Homo sapiens* Crm1cDNA clone  (Open Biosystems). | This study | |
| pET19b-mCrm1 | Amplified from pCDNA-myc-murCrm1 from M. Malim^16^ | This study | |
| pET19b-hRan | Amplified from pQE30-hRan from I. Mattaj^22^ | This study | |
| pET19b-hRanQ69L | Site directed mutagenesis of pET19b-hRan | This study | |
| pBluescript-5’∆RRE | Site directed mutagenesis of pBluescript-SF-2 RRE |  | |
