# Supplementary material for: The export receptor Crm1 forms a dimer to promote nuclear export of HIV RNA
Source: eLife. 2014 Dec 8;3:e04121. doi: 10.7554/eLife.04121 (PMC4360530; doi:10.7554/eLife.04121)
Supplement: Supplementary file 2. — Primers. DOI: http://dx.doi.org/10.7554/eLife.04121.014 [file elife04121s002.docx]

| **Primers** | |
| --- | --- |
| **Name** | **Sequence (5’-3’)** |
| Crm1  Forward | GCACTGACATATGCCAGCAATTATGACAATGTTAGCAGACCATG |
| Crm1  Reverse | CTTCTCGAGTTAATCACACATTTCTTCTGGAATCTCATGTGGATTAAAG |
| Ran  Forward | GCACTGACATATGACCGCGCAGGGAGAG |
| Ran  Reverse | CTTCTCGAGTTACAGGTCATCATCCTCATCCGGGAG |
| RevM10 | GTGCCTCTTCAGCTACCACCGGATCTGAGACTTACTCTTGATTG |
| RanQ69L | GTATGGGACACAGCCGGCCTGGAGAAATTCGGTGGACTG |
| 5’∆RRE | GCGTAATACGACTCACTATAGGGCTAGTAGGAGCTATGTTCC |
